# Live Bird Markets in Nigeria: A Potential Reservoir for H9N2 Avian Influenza Viruses

**DOI:** 10.3390/v13081445

**Published:** 2021-07-24

**Authors:** Lanre Sulaiman, Ismaila Shittu, Alice Fusaro, Bitrus Inuwa, Bianca Zecchin, Dorcas Gado, Alessia Schivo, Alice Bianco, Agnes Laleye, Federica Gobbo, Columba Vakuru, Tony Joannis, Isabella Monne, Clement Meseko

**Affiliations:** 1 National Veterinary Research Institute, Vom 930010, Nigeria; laprecieux@gmail.com (L.S.); usfilmalgwi@yahoo.com (B.I.); dbarms2g@yahoo.com (D.G.); agnesoladokun@yahoo.com (A.L.); tmjoannis@yahoo.com (T.J.); cameseko@yahoo.com (C.M.); 2 Istituto Zooprofilattico Sperimentale delle Venezie, 35020 Legnaro, Italy; afusaro@izsvenezie.it (A.F.); bzecchin@izsvenezie.it (B.Z.); aschivo@izsvenezie.it (A.S.); abianco@izsvenezie.it (A.B.); fgobbo@izsvenezie.it (F.G.); imonne@izsvenezie.it (I.M.); 3 Federal Ministry of Agriculture and Rural Development, Abuja 900246, Nigeria; vakurucolteru@yahoo.com

**Keywords:** avian influenza H9N2, live bird markets, Nigeria, multiple introductions, poultry

## Abstract

Since 2006, multiple outbreaks of avian influenza (AI) have been reported in Nigeria involving different subtypes. Surveillance and molecular epidemiology have revealed the vital role of live bird markets (LBMs) in the dissemination of AI virus to commercial poultry farms. To better understand the ecology and epidemiology of AI in Nigeria, we performed whole-genome sequencing of nineteen H9N2 viruses recovered, from apparently healthy poultry species, during active surveillance conducted in nine LBMs across Nigeria in 2019. Analyses of the HA gene segment of these viruses showed that the H9N2 strains belong to the G1 lineage, which has zoonotic potential, and are clustered with contemporary H9N2 identified in Africa between 2016 and 2020. We observed two distinct clusters of H9N2 viruses in Nigeria, suggesting different introductions into the country. In view of the zoonotic potential of H9N2 and the co-circulation of multiple subtypes of AI virus in Nigeria, continuous monitoring of the LBMs across the country and molecular characterization of AIVs identified is advocated to mitigate economic losses and public health threats.

## 1. Introduction

Since the first incursion of Highly Pathogenic Avian Influenza (HPAI) virus subtype H5N1 into Nigeria in 2006 [1], surveillance and laboratory capacities for Influenza diagnostics have been enhanced in-country. Live bird markets (LBMs), which are proverbial melting pots for different types of poultry species from different sources and states of the country, have been the usual targets for surveillance activities. In addition, available data on HPAI in Nigeria in the last ten years showed the importance of LBMs in the ecology and epidemiology of avian influenza [2]. Furthermore, it is not uncommon to find free-range poultry from households scavenging for food close to or in the LBMs [3], contributing to making LBMs a hotspot for the introduction and spread of emerging Avian Influenza (AI) virus strains to commercial farms. Poor infrastructures, lax biosecurity monitoring and control measures in most LBMs also increase the likelihood of co-circulation of different avian influenza subtypes [4].

Following the first episode of HPAI in Nigeria, several subtypes and reassortant strains of AI including H5N1 [5], H5N2 [6], H5N8 [7,8] and more recently H5N6 [9] have been detected in LBMs across the country but no evidence yet of the presence of H9N2 in Nigeria. However, seroconversion to the H9 subtype has been reported in poultry sampled at LBMs in a limited study in Southwestern Nigeria [10,11]. The low pathogenic avian influenza (LPAI) subtype H9N2 belonging to the G1 lineage has been identified in several countries and has attained endemic status in poultry populations across Asia, Middle East and Northern Africa [12]. 

In West Africa, the first report of the H9N2 subtype was reported in Burkina Faso in 2016 [13], shortly thereafter in Ghana [14], Benin Republic and Togo [15]. An increasing number of H9N2 infections in humans have been reported in the world [12]. In Africa, five human cases have been reported since 2015, four in Egypt and one in Senegal [16]. Together with its zoonotic potential, the circulation of this subtype with other avian influenza virus subtypes is of great concern considering the risk of the emergence of novel reassortant viruses with unpredictable biological properties. Here, we report the molecular characterization of H9N2 viruses recovered during active surveillance conducted in LBMs in Nigeria in 2019.

## 2. Materials and Methods

### 2.1. Viruses

In 2019, active surveillance activity was carried out in LBMs across 18 out of the 36 States including the Federal Capital Territory (Abuja), Nigeria. A total of 3131 tracheal (*n* = 1566) and cloacal (*n* = 1565) samples from 13 bird species were collected by field officers of the Veterinary and Pest Control Services Department, Federal Ministry of Agriculture and Rural Development, Abuja. The samples were collected using polyester cotton swab into viral transport medium and analyzed at National Veterinary Research Institute (NVRI), Nigeria for avian influenza viruses as described previously [8,9].

In the framework of the World Organisation for Animal Health (OIE) twinning project between the NVRI, Nigeria and the Istituto Zooprofilattico Sperimentale delle Venezie (IZSVe), Italy, nineteen isolates (*n* = 8) and diagnostic samples (*n* = 11) of the H9N2 subtype obtained from 9 states in Nigeria during the active surveillance were submitted to IZSVe (Legnaro, Italy) for genetic characterization. The details of the isolates are shown in Table 1.

### 2.2. Genome Amplification and Sequencing 

The whole-genome sequencing was conducted on the H9N2 viruses using the Illumina MiSeq platform. Briefly, total RNA was purified from positive samples using the QIAsymphony DSP Virus/Pathogen Midi kit (Qiagen, Hilden, Germany). The complete genome was amplified by using the SuperScript III One-Step RT-PCR System with Platinum Taq High Fidelity (Invitrogen, Carlsbad, CA, USA) and 1 pair of primers complementary to the conserved elements of the influenza A virus promoter as previously described [7]. The sequencing library was prepared by using the Nextera DNA XT Sample preparation kit (Illumina, San Diego, CA, USA) and quantified by using the Qubit dsDNA High Sensitivity Kit (Invitrogen, Carlsbad, CA, USA). The High Sensitivity DNA Analysis Kit (Agilent Technologies, Alpharetta, GA, USA) was used to determine the average fragment length. The indexed libraries were pooled in equimolar concentrations and sequenced in multiplex for 250 bp paired-end on Illumina MiSeq, according to the manufacturer’s instructions.

### 2.3. Illumina Sequencing Data Analysis

FastQC version 0.11.2 (https://www.bioinformatics.babraham.ac.uk/projects/fastqc/ accessed on 16 December 2019) was used to assess read quality. Raw data were filtered by removal of reads with >10% of undetermined bases, reads with >100 bases with a Q score of 80 bases were aligned against a reference genome by using BWA version 0.7.12 (20). Picard-tools version 2.1.0 (http://picard.sourceforge.net/ accessed on 16 December 2019) and GATK version 3.5 [17,18] were used to correct potential errors, realign reads around indels, and recalibrate base quality. LoFreq version 2.1.2 [19] was used to call single-nucleotide polymorphisms. Outputs were used to generate consensus sequences. Gene sequences of the 19 isolates generated in this study were deposited in GenBank under the accession numbers MZ150582–MZ150733.

### 2.4. Phylogenetic Analyses 

Consensus sequences of each gene segment of the Nigerian virus were compared with the most related sequences available in GISAID reported in Technical Appendix Table 1 (https://www.gisaid.org/ accessed on 9 April 2021) and aligned by using MAFFT version 7 [20]. IQTREE version 1.6 was used to construct the maximum likelihood phylogenetic trees applying the best-fit general time-reversible model of nucleotide substitution with gamma-distributed rate variation among sites (GTR + F + I + G4) and performing ultrafast bootstrap resampling analysis (1000 replications) [21]. Phylogenetic trees were visualized by using FigTree version 1.4.2 (http://tree.bio.ed.ac.uk/software/figtree/ accessed on 9 April 2021).

### 2.5. Bayesian Analysis

The hemagglutinin (HA) gene sequences were used to perform a Markov chain Monte Carlo (MCMC) analysis in BEAST v10.4 [22]. A HKY85 + Γ4 model of nucleotide substitution with two data partitions of codon positions (1st + 2nd positions, 3rd position) was adopted, with base frequencies unlinked across all codon positions (SRD06 substitution model). We used a relaxed uncorrelated lognormal molecular clock and a Skyride coalescent model. Chain lengths were run for 30 million iterations to achieve convergence as assessed using Tracer v1.6 (http://beast.bio.ed.ac.uk/Tracer/ accessed on 9 April 2021). TreeAnnotator v1.10.4 [22] was used to generate the Maximum Clade Credibility (MCC) phylogenetic tree, adopting an appropriate burn-in (10% of the trees). The MCC tree was visualized in FigTree v1.4.2 (http://tree.bio.ed.ac.uk/software/figtree/ accessed on 9 April 2021) in order to identify the tMRCA (time to the most recent common ancestor).

## 3. Results

**Figure 1:**
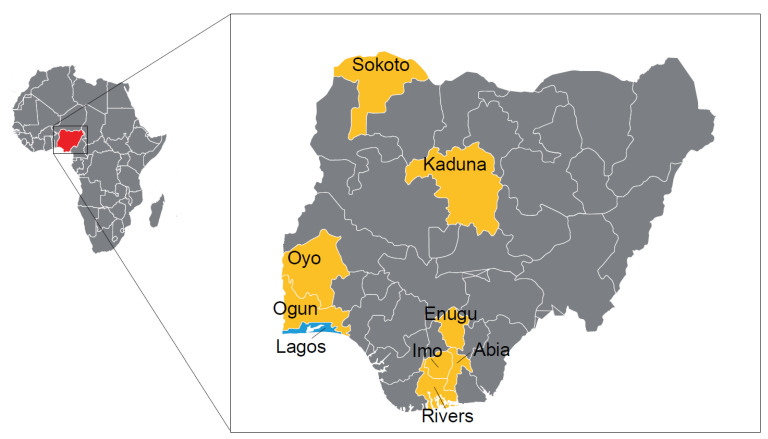
A map of Nigeria showing all the states including the Federal Capital Territory (FCT). The locations where the H9N2 viruses were obtained are highlighted in yellow (cluster I) and blue (cluster II).

**Figure 2:**
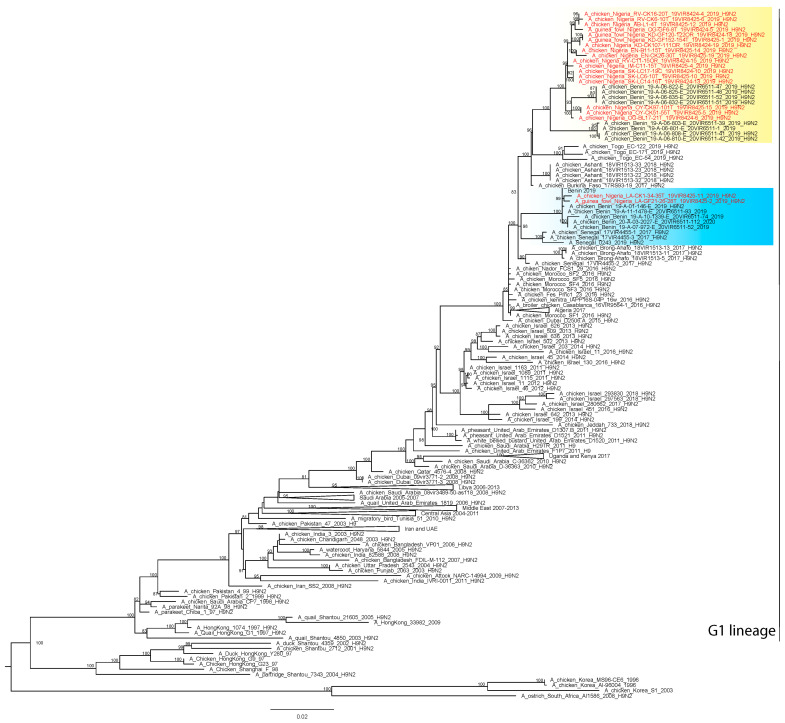
Maximum Likelihood phylogenetic tree of the HA gene (IQ-TREE v.1.6.8). The H9N2 viruses from Nigeria are marked in red. Ultra-fast bootstrap supports higher than 80% are indicated next to the nodes.

The complete genome of the H9N2 viruses (n = 19) obtained from apparently healthy poultry species (chickens and guinea fowl) in LBMs from the nine states namely: Rivers (*n* = 3); Sokoto (*n* = 3); Kaduna (*n* = 3); Ogun (*n* = 2); Lagos (*n* = 2), Oyo (*n* = 2); Enugu (*n* = 2); Imo (*n* = 1); and Abia (*n* = 1) (Figure 1) were successfully sequenced. Analyses of the complete HA gene segment of these viruses showed that the H9N2 strains belong to the G1 lineage, which has zoonotic potential. In particular, the viruses clustered with contemporary H9N2 subtype viruses identified in North (Morocco and Algeria) and West Africa (Burkina Faso; Benin Republic, Togo, Senegal and Ghana) between 2016 and 2020 (Figure 2).

The H9N2 viruses from Nigeria fell within two distinct clusters co-circulating in West Africa, suggesting the occurrence of different introductions into the country. Specifically, the two viruses collected in Lagos State (A/guinea_fowl/Nigeria/LA-GF21-26-28T_19VIR8425-2/2019 and A/chicken/Nigeria/LA-CK1-34-35T_19VIR8425-11/2019) fall within a cluster together with viruses collected in the Benin Republic in 2019-2020; all the Nigerian H9N2 viruses (Figure 2) from the other eight states belong to a separate cluster and are closely related to viruses collected in the Benin Republic in 2019. This clustering was confirmed by the analyses of the remaining genes (supplementary data, Figures S1–S8). 

The cleavage site of the H9N2 viruses from Lagos State differs by one amino acid (KSSR/GLF) from the cleavage site of the other Nigerian H9N2 viruses (RSSR/GLF). All the viruses possess the mammalian adaptation markers I155T and Q226L (H3 numbering) in the HA gene which are the mutations promoting the preferential binding to human-like α2-6-linked sialic acid receptors.

In regards to the NA gene segment, Nigerian viruses showed a potential additional glycosylation site at position 329-331 (NDS) and the loss of two glycosylation sites at positions 69-71 and 86-88, as shown by the related H9N2 viruses identified in North and West Africa between 2016 and 2018 and in some H9N2 from the Middle Eastern countries. From the Bayesian analysis, the most recent common ancestor of the Nigerian strains collected in Lagos emerged between January-June 2019, while the introduction of the H9N2 virus in the other States likely occurred over the period June 2018–March 2019. These results are constrained by the availability of a limited number of sequences from Western African region. Therefore, it cannot be excluded that the introductions have occurred from countries for which there are no data.

## 4. Discussion

In this study, we reported, for the first time, AIV H9N2 belonging to lineage G1 from apparently healthy poultry sold in nine LBMs across Nigeria. The first report of the low pathogenic avian influenza subtype H9N2 lineage in West Africa was in Burkina Faso [13] this report came 29 years after the subtype was detected in China [23]. 

An inferred diverse introduction of H9N2 into Nigeria is consistent with previous reports of multiple introductions of avian influenza into Nigeria since 2006 [24]. On one hand, the clustering observed in the two isolates from Lagos with those from the Benin Republic and on the other, the clustering of the H9N2 viruses from the other eight states with the Benin Republic, Togo and Ghana may suggest trade-related introduction. It has been suggested that legal and illegal poultry trade, as well as wild bird migrations, contribute to influenza virus spread into West Africa, with Nigeria acting as a crucial hotspot for virus introduction and dissemination into the continent [7]. Therefore, the risk of introduction and re-introduction of novel influenza virus from infected regions in Asia and Europe as well as bordering regions is higher in Nigeria given the large geographical space, poultry density and abundance of wetland. Bridging this introduction of avian influenza subtypes such as H9N2 is the LBM which further distribute infection to commercial poultry. While little is known of H9N2 infection in commercial poultry in Nigeria, an earlier report from Ghana described the potential for H9N2 to complicate respiratory viral infections and may cause unusually high morbidity and mortality [14]. Additionally significant is the zoonotic potential of the H9N2 isolates from Nigeria as they harbor the mammalian adaptation markers I155T and Q226L (H3 numbering) in the HA gene, which promote preferential binding to human-like α2-6-linked sialic acid receptors [25]. These mutations have been reported in H9N2 found in other African countries [13,14,16]. Silent circulation of H9N2 with less than optimum biosecurity makes the virus more likely to be transmitted to other species akin to the observation by Meseko et al. [26] where H5N1 was transmitted to domestic pigs. This may further complicate the multiplicity of strains, reassortment and emergence of novel strains with unpredictable phenotypic implications.

In view of the above, improvement of the monitoring systems of these viruses in farms and hotspots like the LBMs is needed. Continuous education of and collaboration with–LBM operators is also advocated as they can play a major role in the early detection and control of Avian Influenza viruses.

## 5. Conclusions

The outbreaks of H9N2 viruses have been on the increase in several continents and may be causing greater economic damage to poultry production worldwide than we currently realize. The possibilities of reassortant H9N2-origin viruses as a result of co-circulation with other Influenza viruses are currently high in Nigeria. All these facts indicate a growing threat from H9N2 viruses to both animal and human health. Nigeria, having gone through several Avian Influenza epidemics, cannot afford to be caught unaware by another outbreak as the economic loss will exacerbate the current situation. Having mentioned the zoonotic potential of H9N2 viruses, Nigeria would also not want to add that burden to the current public health challenge occasioned by the COVID-19 pandemic, adding to pre-existing human health challenges of the country. It is therefore imperative to put resources in place for the continuous monitoring of these Influenza viruses as a basis for pro-active intervention for the control and possible eradication of these viruses.

## Figures and Tables

**Table 1:** Epidemiological information of the Nigerian H9N2 isolates used in this study.

S/N ^#^	Isolates ID	Scientific Name	Location	Collection Date
1	A/chicken/Nigeria/RV-CK16-20T_19VIR8424-4/2019	Gallus domesticus	Rivers	7 May 2019
2	A/chicken/Nigeria/RV-CK6-10T_19VIR8425-6/2019	Gallus domesticus	Rivers	19 July 2019
3	A/chicken/Nigeria/RV-C11-15OR_19VIR8424-15/2019	Gallus domesticus	Rivers	7 December 2019
4	A/chicken/Nigeria/SK-LC17-19C_19VIR8424-10/2019	Gallus domesticus	Sokoto	7 July 2019
5	A/chicken/Nigeria/SK-LC14-16T_19VIR8424-13/2019	Gallus domesticus	Sokoto	7 July 2019
6	A/chicken/Nigeria/SK-LC6-10T_19VIR8425-10/2019	Gallus domesticus	Sokoto	20 July 2019
7	A/guinea_fowl/Nigeria/KD-GF152-154T_19VIR8425-1/2019	Numida meleagris	Kaduna	17 July 2019
8	A/guinea_fowl/Nigeria/KD-GF120-122OR_19VIR8424-18/2019	Numida meleagris	Kaduna	7 November 2019
9	A/chicken/Nigeria/KD-CK107-111OR_19VIR8424-19/2019	Gallus domesticus	Kaduna	7 November 2019
10	A/guinea_fowl/Nigeria/LA-GF21-26-28T_19VIR8425-2/2019	Numida meleagris	Lagos	18 July 2019
11	A/chicken/Nigeria/LA-CK1-34-35T_19VIR8425-11/2019	Gallus domesticus	Lagos	25 July 2019
12	A/chicken/Nigeria/EN-CK26-30T_19VIR8425-19/2019	Gallus domesticus	Enugu	8 April 2019
13	A/chicken/Nigeria/EN-B11-15T_19VIR8425-14/2019	Gallus domesticus	Enugu	28 July 2019
14	A/guinea_fowl/Nigeria/OG-GF6-8T_19VIR8424-5/2019	Numida meleagris	Ogun	7 May 2019
15	A/chicken/Nigeria/OG-BL17-21T_19VIR8424-6/2019	Gallus domesticus	Ogun	7 May 2019
16	A/chicken/Nigeria/OY-CK97-101T_19VIR8425-15/2019	Gallus domesticus	Oyo	8 April 2019
17	A/chicken/Nigeria/OY-CK51-55T_19VIR8425-5/2019	Gallus domesticus	Oyo	26 July 2019
18	A/chicken/Nigeria/AB-L1-4T_19VIR8425-12/2019	Gallus domesticus	Abia	23 July 2019
19	A/chicken/Nigeria/IM-C11-15T_19VIR8425-4/2019	Gallus domesticus	Imo	19 July 2019

^#^ Serial number.

## Data Availability

Gene sequences of the 19 isolates generated in this study were deposited in GenBank under the accession numbers MZ150582–MZ150733.

## Supplementary Materials

The following are available online at https://www.mdpi.com/article/10.3390/v13081445/s1, Figure S1: Maximum Likelihood phylogenetic tree of the NA gene (IQ-TREE v.1.6.8). The H9N2 viruses from Nigeria are marked in red. Ultra-fast bootstrap supports higher than 80% are indicated next to the nodes; Figure S2. Maximum Likelihood phylogenetic tree of the PB2 gene (IQ-TREE v.1.6.8). The H9N2 viruses from Nigeria are marked in red. Ultra-fast bootstrap supports higher than 80% are indicated next to the nodes; Figure S3: Maximum Likelihood phylogenetic tree of the PB1 gene (IQ-TREE v.1.6.8). The H9N2 viruses from Nigeria are marked in red. Ultra-fast bootstrap supports higher than 80% are indicated next to the nodes; Figure S4: Maximum Likelihood phylogenetic tree of the PA gene (IQ-TREE v.1.6.8). The H9N2 viruses from Nigeria are marked in red. Ultra-fast bootstrap supports higher than 80% are indicated next to the nodes; Figure S5: Maximum Likelihood phylogenetic tree of the NP gene (IQ-TREE v.1.6.8). The H9N2 viruses from Nigeria are marked in red. Ultra-fast bootstrap supports higher than 80% are indicated next to the nodes; Figure S6: Maximum Likelihood phylogenetic tree of the M gene (IQ-TREE v.1.6.8). The H9N2 viruses from Nigeria are marked in red. Ultra-fast bootstrap supports higher than 80% are indicated next to the nodes; Figure S7: Maximum Likelihood phylogenetic tree of the NS gene (IQ-TREE v.1.6.8). The H9N2 viruses from Nigeria are marked in red. Ultra-fast bootstrap supports higher than 80% are indicated next to the nodes; Figure S8: MCC tree of HA of H9N2 viruses identified in Nigeria.
