# Supplementary figures and images for: Live Bird Markets in Nigeria: A Potential Reservoir for H9N2 Avian Influenza Viruses
Source: Viruses. 2021 Jul 24;13(8):1445. doi: 10.3390/v13081445 (PMC8402768; doi:10.3390/v13081445)

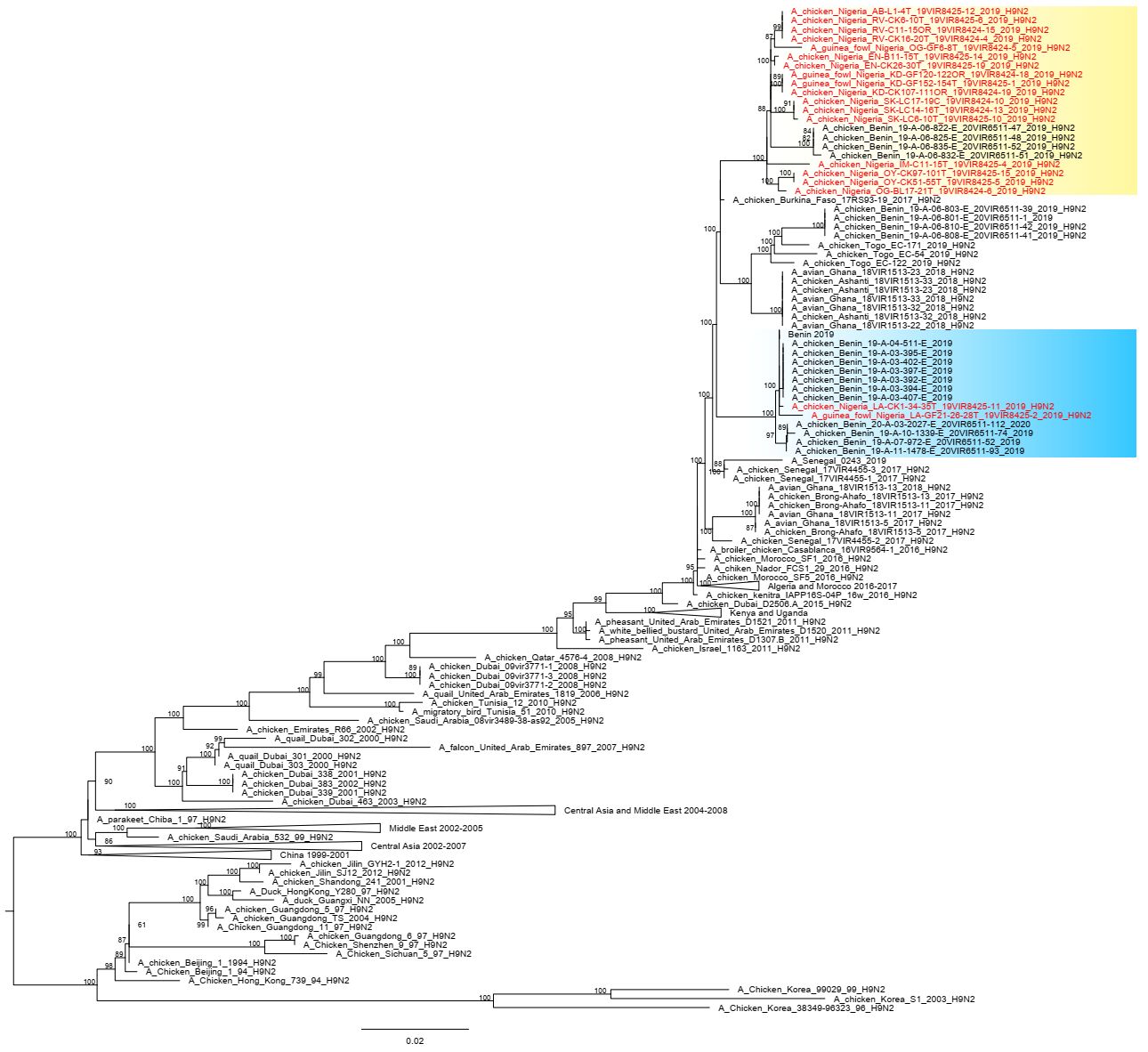

Supplement: Supplementary file 1 [file viruses-13-01445-s001.zip › supporting document_H9N2 Nigeria/SF1- NA.jpg]

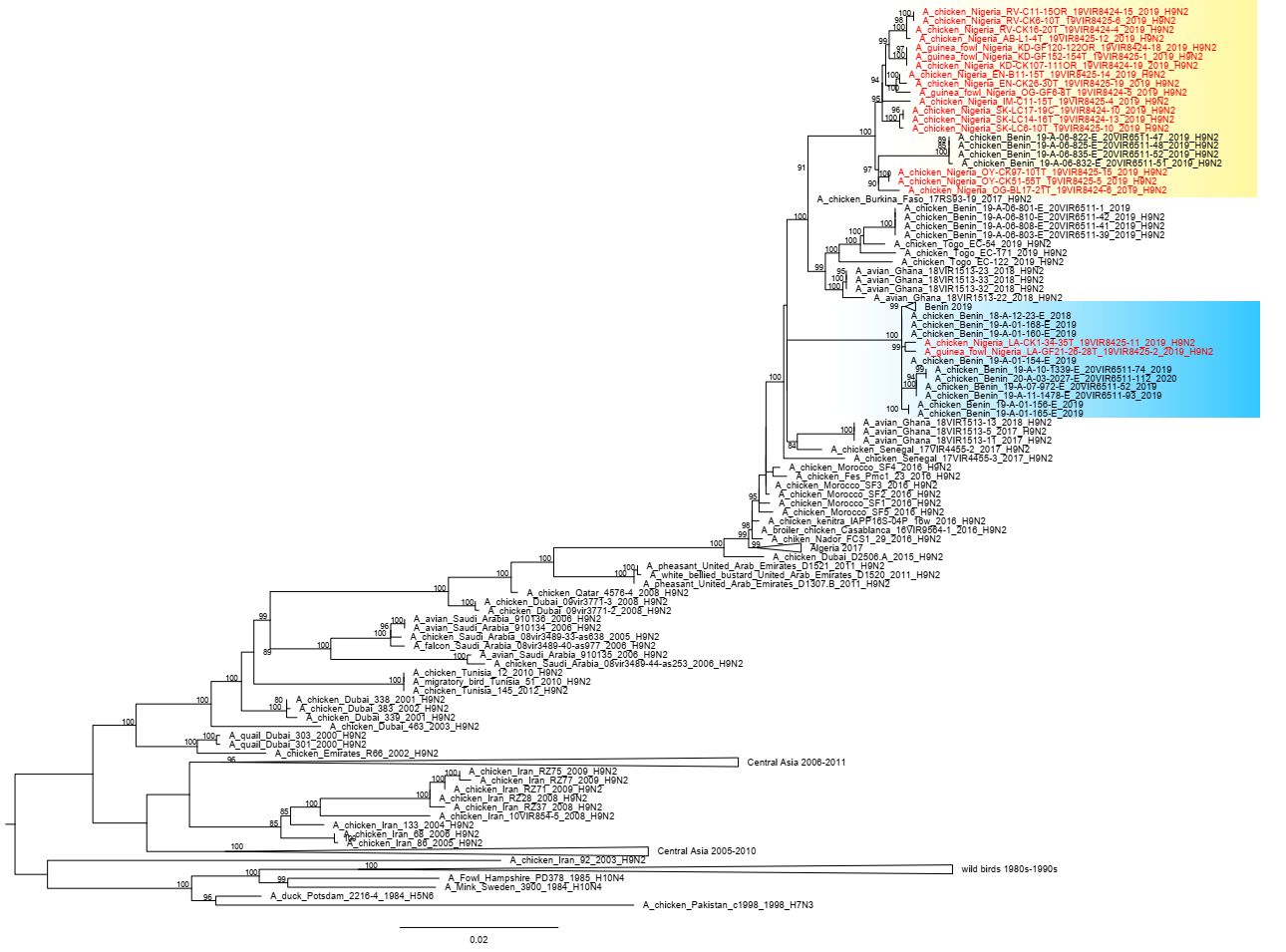

Supplement: Supplementary file 1 [file viruses-13-01445-s001.zip › supporting document_H9N2 Nigeria/SF2 - PB2.jpg]

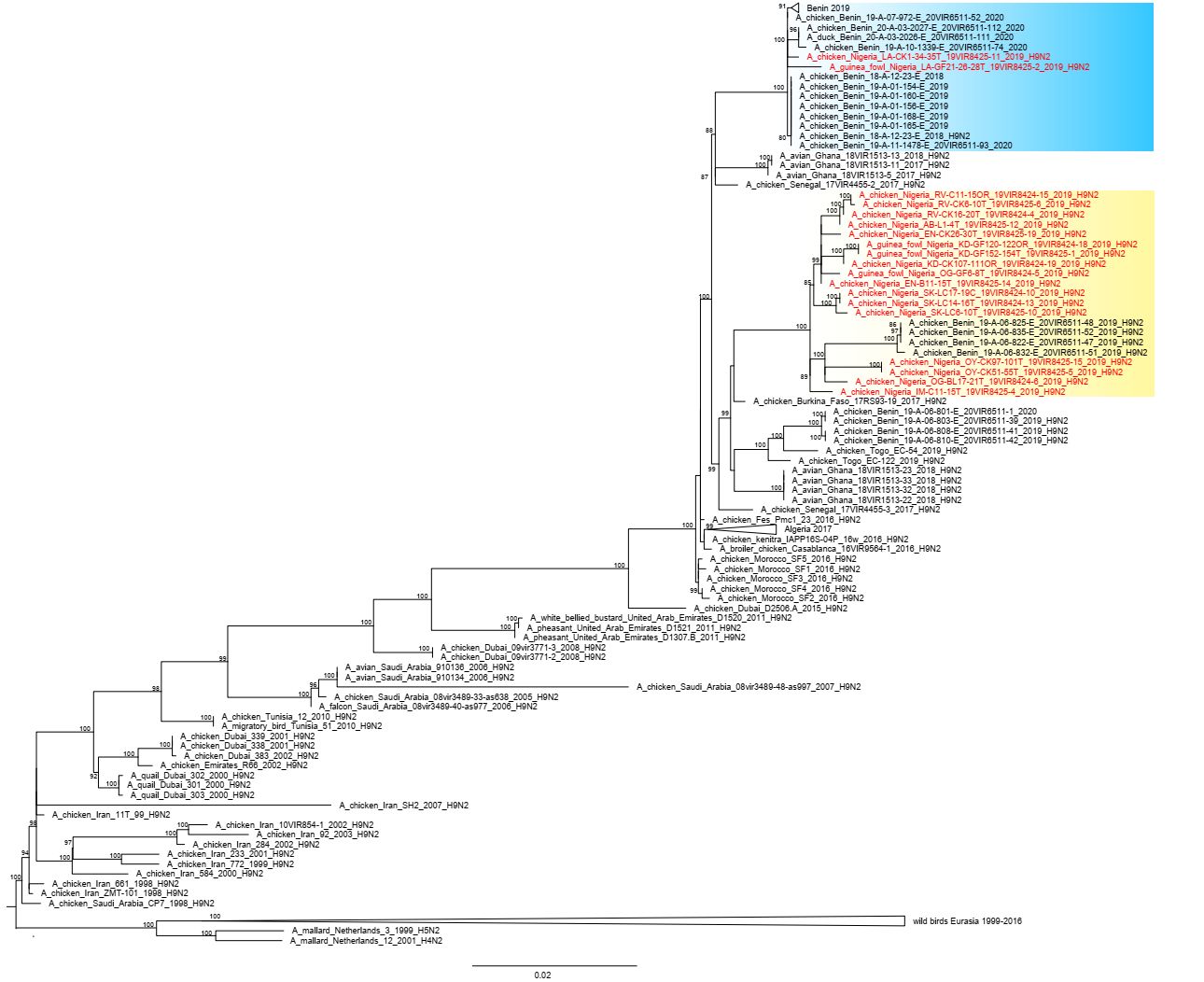

Supplement: Supplementary file 1 [file viruses-13-01445-s001.zip › supporting document_H9N2 Nigeria/SF3 - PB1.jpg]

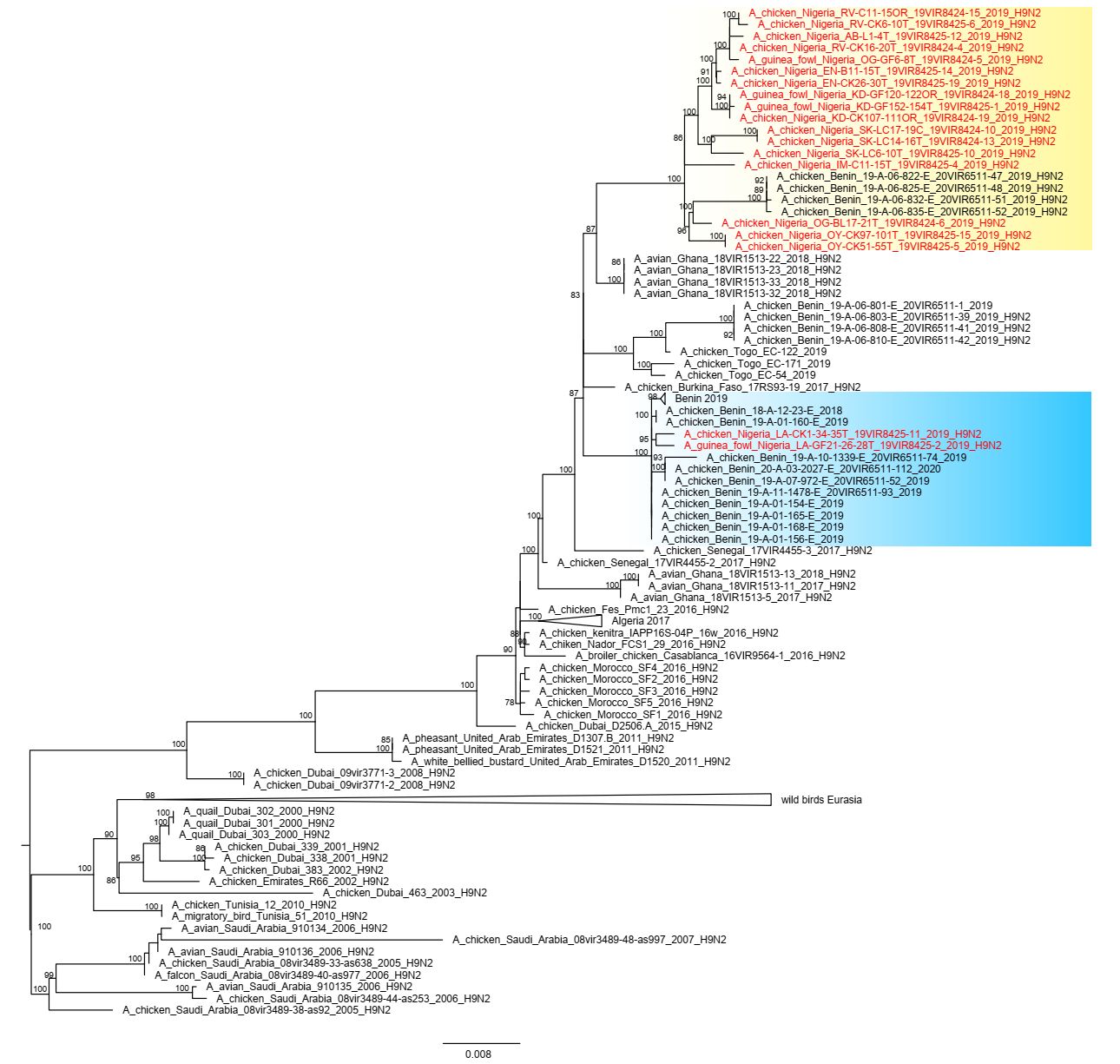

Supplement: Supplementary file 1 [file viruses-13-01445-s001.zip › supporting document_H9N2 Nigeria/SF4 - PA.jpg]

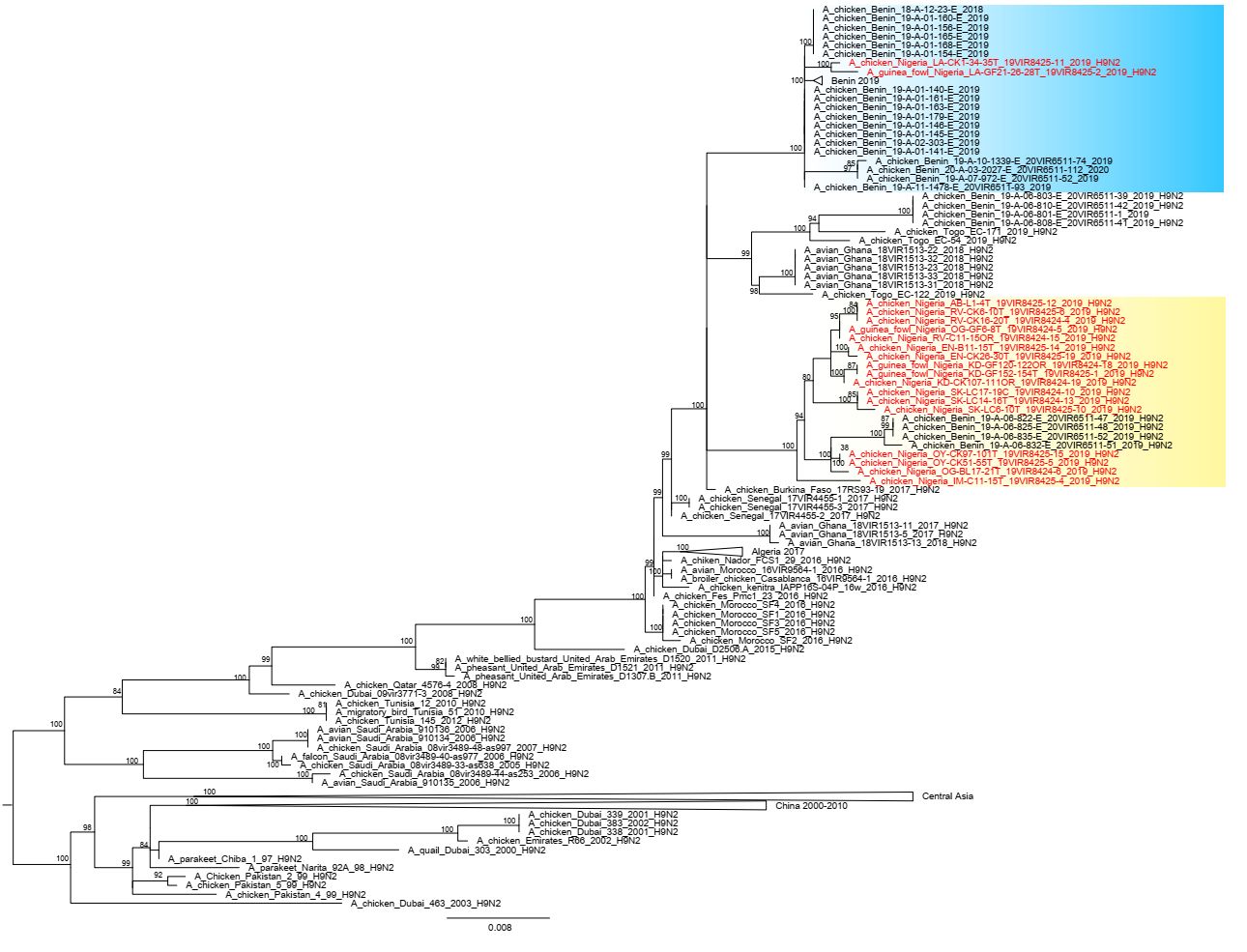

Supplement: Supplementary file 1 [file viruses-13-01445-s001.zip › supporting document_H9N2 Nigeria/SF5 - NP.jpg]

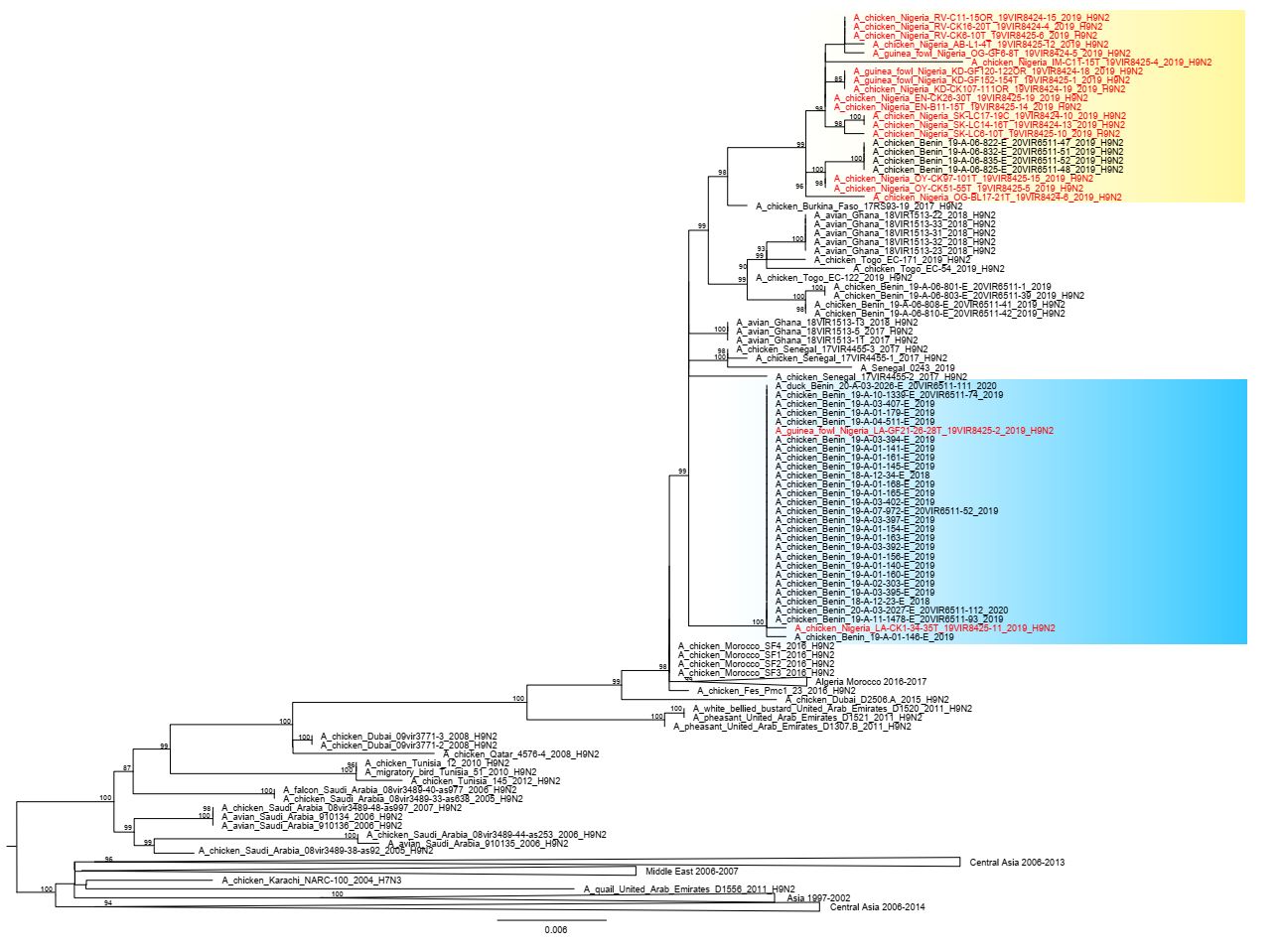

Supplement: Supplementary file 1 [file viruses-13-01445-s001.zip › supporting document_H9N2 Nigeria/SF6 - M.jpg]

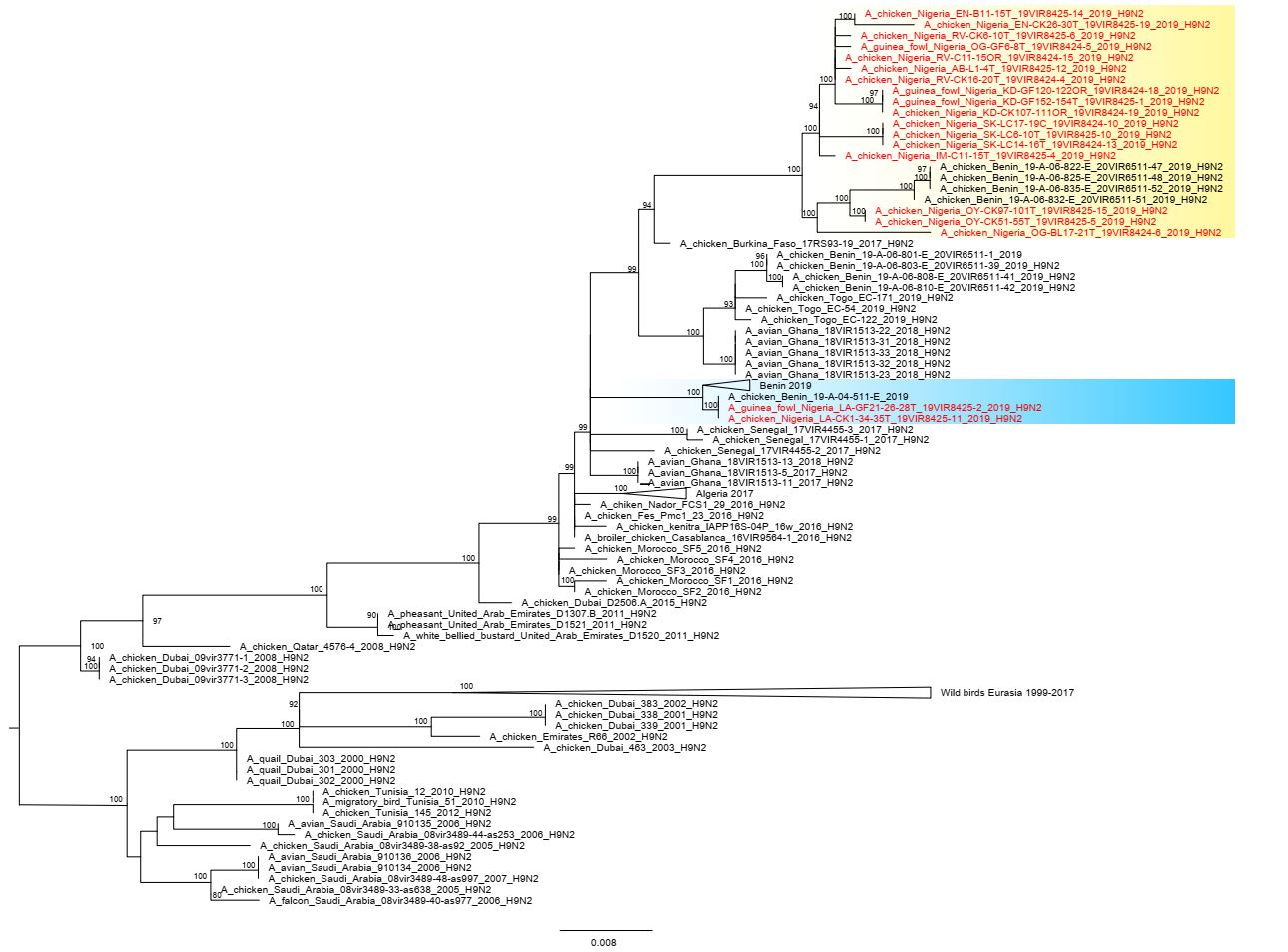

Supplement: Supplementary file 1 [file viruses-13-01445-s001.zip › supporting document_H9N2 Nigeria/SF7 - NS.jpg]

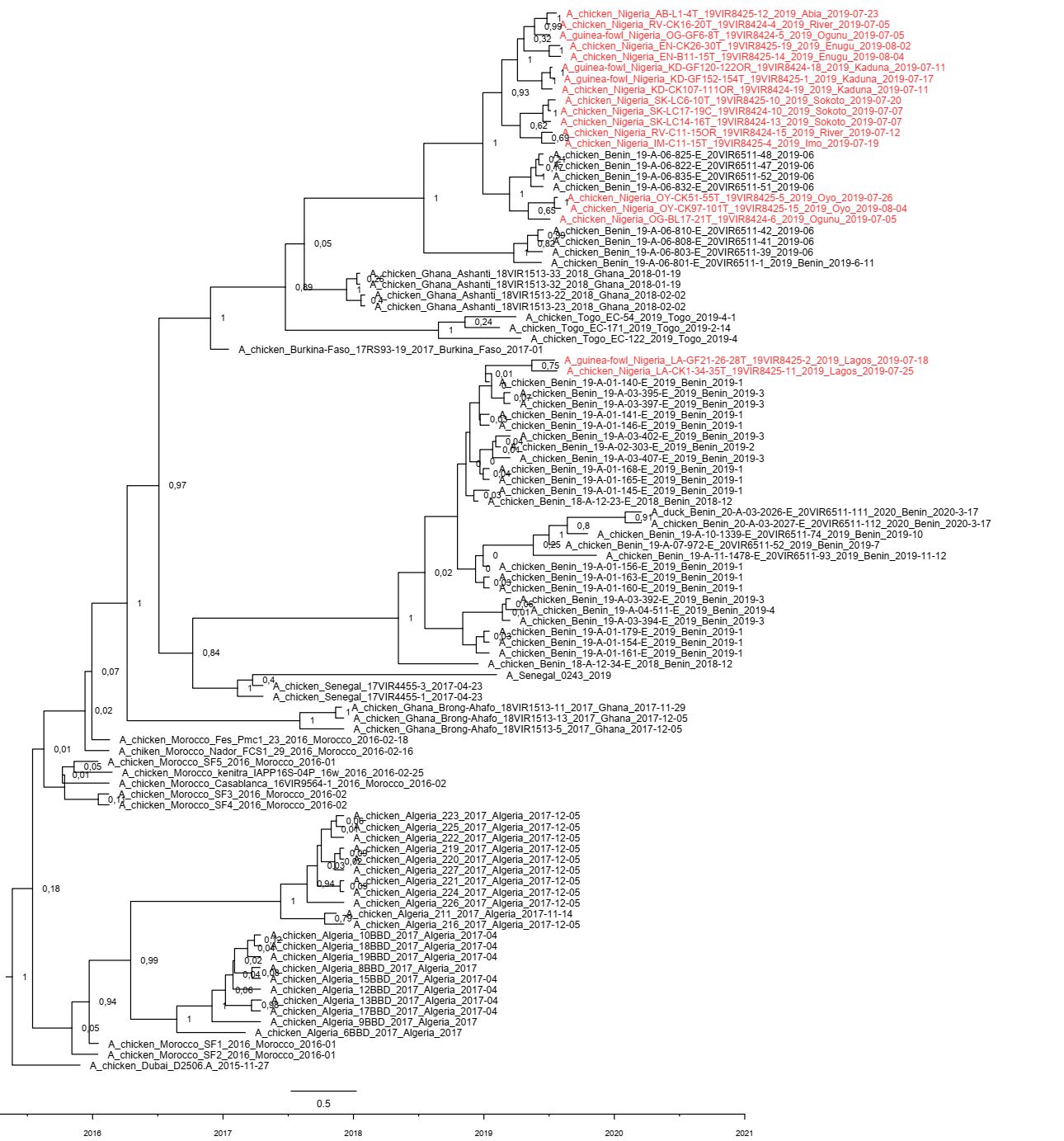

Supplement: Supplementary file 1 [file viruses-13-01445-s001.zip › supporting document_H9N2 Nigeria/SF8 - MCC Tree.jpg]
